# Disordered Sleep and Myopia Risk among Chinese Children

**DOI:** 10.1371/journal.pone.0121796

**Published:** 2015-03-26

**Authors:** Zhongqiang Zhou, Ian G. Morgan, Qianyun Chen, Ling Jin, Mingguang He, Nathan Congdon

**Affiliations:** 1 State Key Laboratory of Ophthalmology and Division of Preventive Ophthalmology, Zhongshan Ophthalmic Center, Sun Yat-sen University, Guangzhou, China; 2 ARC Centre of Excellence in Vision Science, Research School of Biology, Australian National University, Canberra, Australia; 3 ORBIS International, New York, New York, United States of America; Sun Yat-sen University, CHINA

## Abstract

**Purpose:**

Disordered sleep and myopia are increasingly prevalent among Chinese children. Similar pathways may be involved in regulation of both sleep cycles and eye growth. We therefore sought to examine the association between disordered sleep and myopia in this group.

**Methods:**

Urban primary school children participating in a clinical trial on myopia and outdoor activity underwent automated cycloplegic refraction with subjective refinement. Parents answered questions about children's sleep duration, sleep disorders (Children's Sleep Habits Questionnaire [CSHQ]), near work and time spent outdoors.

**Results:**

Among 1970 children, 1902 (96.5%, mean [standard deviation SD] age 9.80 [0.44] years, 53.1% boys) completed refraction and questionnaires. Myopia < = -0.50 Diopters was present in both eyes of 588 (30.9%) children (1329/3804 = 34.9% of eyes) and 1129 children (59.4%) had abnormal CSHQ scores (> 41). In logistic regression models by eye, odds of myopia < = -0.50D increased with worse CSHQ score (Odds Ratio [OR] 1.01 per point, 95% Confidence Interval [CI] [1.001, 1.02], P = 0.014) and more night-time sleep (OR 1.02, 95% CI [1.01, 1.04, P = 0.002], while male sex (OR 0.82, 95% CI [0.70, 0.95], P = 0.008) and time outdoors (OR = 0.97, 95% CI [0.95, 0.99], P = 0.011) were associated with less myopia. The association between sleep duration and myopia was not significant (p = 0.199) for total (night + midday) sleep.

**Conclusions:**

Myopia and disordered sleep were both common in this cohort, but we did not find consistent evidence for an association between the two.

**Trial Registration:**

clinicaltrials.gov NCT00848900

## Introduction

Chinese children have been shown to have a high prevalence of myopia [1,2], which has increased over the past few decades. [3] This has led to much interest in environmental factors that might explain increasing myopia among children of Chinese ethnicity; increased exposure to near work [4–8] and reduced time spent out of doors [8, 9–14] have been suggested as possible candidates.

Recent studies suggest that Chinese children sleep less at night than their peers in the United States and elsewhere [15, 16] and may have a higher prevalence of sleep disorders. [15] The problem of poor sleep may actually be worsening among Chinese children. [17, 18] Poor sleep has been associated with a number of other conditions including attention deficit disorder,[19] overweight and obesity, [20] injuries, [21] poor academic performance [22] and dry eye [23] among Chinese children. An acknowledged flaw in some sleep research on Chinese children has been the failure to include midday sleep, [15] which is a common practice in China, and almost universal for primary school age children.

There is considerable overlap between the biological pathways controlling sleep and ocular development. The circadian cycle in melatonin synthesis and release is a fundamental regulator of sleep, and is controlled by reciprocal interactions with retinal dopaminergic pathways. [24, 25] These dopaminergic pathways also appear to be involved in the regulation of eye growth in humans, and disruptions in circadian rhythms have been reported in both human and animal studies of ocular growth. [24, 25] The goal of the current manuscript is to examine possible cross-sectional associations between prevalent myopia and parentally-reported sleep duration and sleep disorders as assessed using a standard instrument, the Children's Sleep Habits Questionnaire. [26] Night-time and midday sleep duration were ascertained separately. Given the known association between school schedule and poor sleep among Chinese children, [16] we also assessed the amount of time spent in school-related activities in this cohort.

## Methods

The protocol for this study was approved in full by the Institutional Review Board of Zhongshan Ophthalmic Center, Sun Yat-sen University (Guangzhou, China). The clinical trial with which this study was affiliated has been registered at the website clinicaltrials.gov, with the registration number NCT00848900. Permission was received from the local Board of Education, and informed written consent was obtained from at least one parent of all participants. The principles of the Declaration of Helsinki were followed throughout.

Children in the current study were all participants in an on-going randomized trial designed to study the impact of outdoor activity on myopia progression. The Guangzhou Outdoor Activity Longitudinal Study is a cluster-randomized (by school) clinical trial of the effect of increased time outdoors on incident myopia. Twelve public primary schools in Guangzhou, southern China, were allocated to control or an intervention increasing time outdoors by one hour a day, and all children of consenting parents in Grade 1 at baseline were enrolled. This included slightly over 1000 students in each arm at baseline, of whom approximately 900 students in each arm participated in the final examinations after three years. All of these children in the third year of the trial (thus in the fourth grade) are included in the current report.

### Measurement of refractive error

Cycloplegia was induced with a single drop of cyclopentolate 1% after topical anesthesia with one drop of proparacaine hydrochloride 0.5%. A second drop of cyclopentolate was given 5 minutes later, and a third 15 minutes after that if the pupil diameter remained < 6mm and/or if the pupillary reflex was still present. This was sufficient to eradicate the pupillary response in all cases. Automated refraction (Topcon KR 8900,Tokyo, Japan) was carried out five times in each eye. The mean was computed automatically and this value was used as the starting point for subjective refinement by an experienced refractionist. We used the automated cycloplegic value in all calculations in the current report.

### Questionnaire

A questionnaire was given to all children at school to be taken home for completion by their parents. Questions about children’s sleep habits in a “typical” recent week were taken directly from a Chinese-language version [27] of the Children's Sleep Habits Questionnaire, [26] and covered eight subcategories: bedtime resistance, sleep onset delay, sleep duration, sleep anxiety, night waking, parasomnias, sleep disordered breathing and daytime sleepiness. A score of > = 41 points has been suggested to identify children with sleep disorders with adequate levels of sensitivity and specificity. [26] Parents were also asked to document the number of hours spent weekly during the previous year on near work (at <50-cm working distance) including all work in and assigned by regular school, parents, "cram schools," non-academic (eg: art, music) classes and free reading during weekdays, weekends, and holidays. The number of hours spent in night-time sleep at home and midday sleep at school was also estimated by parents.

Finally, the child's total weekly time spent outdoors was recorded by parents, including outdoor activities after getting up but before school, time outdoors during recess and physical education classes, outdoor activities during the midday school break, after leaving school but before sleeping on weekdays, during extracurricular organized sports and sports classes, and on holidays and weekends.

### Statistical Methods

Spherical equivalent refractive error (SER) was calculated as sphere + ½ cylinder, and myopia was defined as SER < = -0.5D in both eyes for the purposes of assessing prevalence, and in single eyes for all analyses by eye. Sleep patterns, CSHQ scale scores, time spent outdoors and near work time were compared between myopic and non-myopic children, with adjustment for school clustering effects, using mixed random effects model for continuous variables with normal distribution, the cluster-adjusted Wilcoxon Rank Sum test as implemented by SAS (stratify.cluswilcox.sas) for non-normally distributed continuous variables, and multilevel mixed-effects logistic regression for binary variables. The association between myopia < = -0.50D in an eye and potential predictors including age, sex, parentally reported sleep duration, score on the CSHQ, hours of near work and time spent outdoors were examined by multilevel mixed-effects logistic models adjusted for clustering within school and between eyes of a child. All statistical analyses were performed using Stata 10.0 (Stata Corp, College Station, TX, USA).

## Results

**Fig 1:**
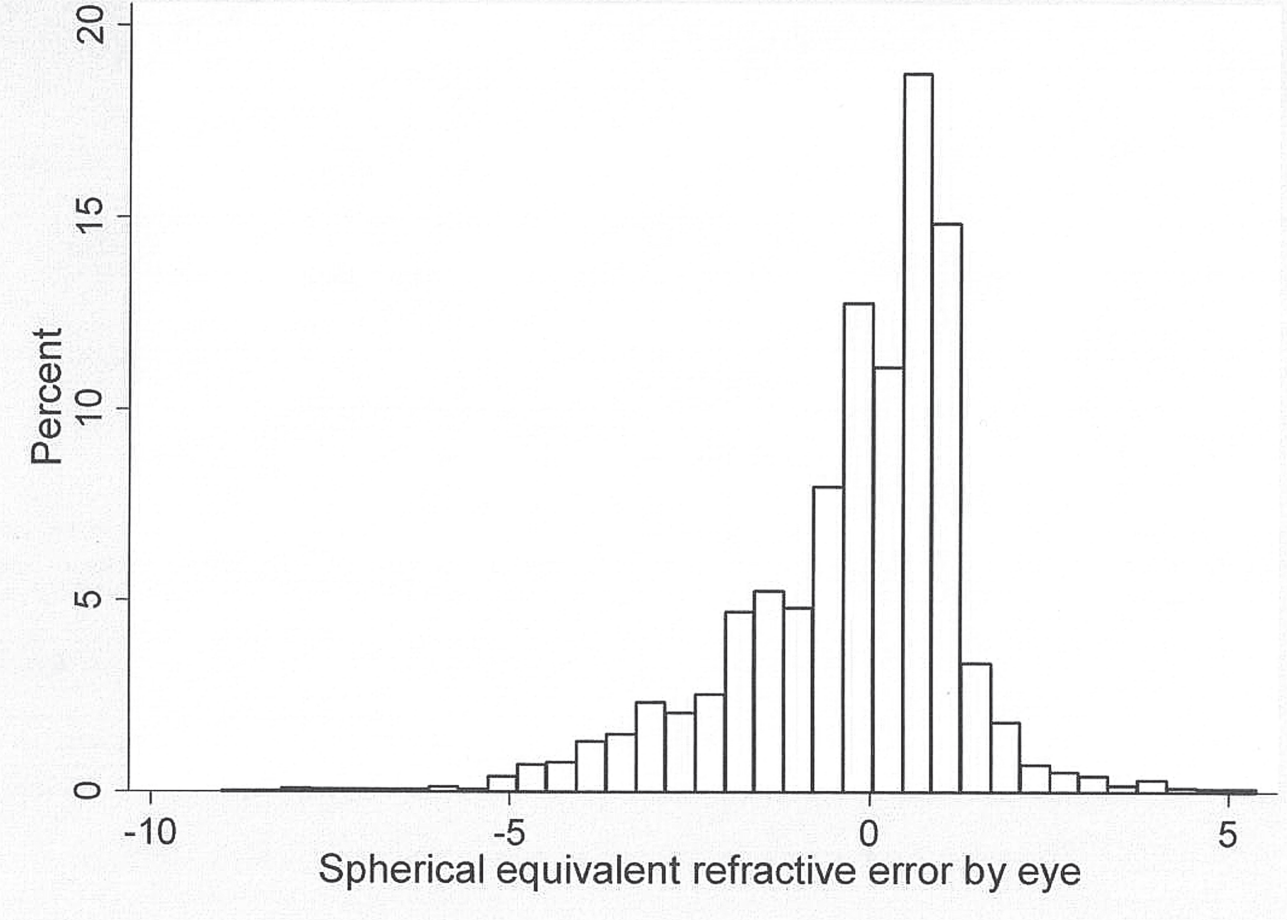
Distribution of spherical equivalent refractive error (Diopters) in 3804 eyes of 1902 children with complete data in the current study

A total of 1970 children were available for study, among whom refraction was carried out in 1961 (99.5%), 1912 (97.1%) had questionnaires returned by their parents and 1902 (96.5%) had complete examination and questionnaire data. The mean age (standard deviation [SD]) of children was 9.80 (0.44) years, and 1010 (53.1%) were boys. (Table 1) Myopia < = -0.50 Diopters was present in both eyes of 588 (30.9%) children, and in 1329 of 3804 eyes (39.4%). The mean (SD) spherical equivalent refractive error for all eyes was -0.05 (1.54) diopters (D), with a range of -8.25 to +.38 D. Fig. 1 shows the distribution of spherical equivalent refractive error for both eyes of children with complete data.

**Table 1:** Basic Demographic Information, Visual Acuity and Spherical Equivalent Refractive error.

	**Characteristic**
**Age (yrs)**	
< = 9	403 (21.2)
10	1460 (76.8)
> = 11	24 (1.26)
Missing	15 (0.79)
Total	1902
Mean (sd), years	9.80 (0.44)
**Sex, N (%)**	
Male	1010 (53.1)
Female	879 (46.2)
Missing	13 (0.68)
Total	1902
**Spherical equivalent refractive error in better-seeing eye (Diopter), N (%)**	
Missing	0
Total	1902
Mean (Standard Deviation)/median(range)	-0.05 (1.54)/0.25(-8.25–+5.38)

Families reported that children with myopia < = -0.5D in both eyes (n = 588) slept a mean (SD) of 9.13 (0.73) hours per night, which was slightly but significantly (at a borderline level) more than was reported for children without myopia in either eye (n = 1314) at 9.06 (0.71) hours (P = 0.052). The proportion of children sleeping an average of < = 8 hours per night did not differ significantly between myopic (114/588 = 19.4%) and non-myopic (279/1314 = 21.3%, P = 0.359) children. Neither the proportion of children with midday sleep (approximately 80%) nor the mean reported duration of midday (approximately an hour/day) or total (10 hours/day) sleep differed between myopic and non-myopic children (P > 0.4 for all). (Table 2)

**Table 2:** Children's Sleep Patterns, Sleep Habits Questionnaire (CSHQ) Scale Scores, Near Work and Time Spent Outdoors, Stratified by Myopia Status.

	**Myopic (< = -0.5D of myopiain both eyes)n = 588**	**Not myopic (> -0.5Dof myopiain either eye)n = 1314**	**P** *
**Night-time sleep duration (hours), n (%)**			0.052**
7	9 (1.53)	14 (1.07)	
8	105 (17.9)	265 (20.2)	
9	282 (48.0)	669 (50.9)	
10	170 (28.9)	335 (25.5)	
> 10	22 (3.74)	30 (2.28)	
Missing	9 (0%)	1 (0.08%)	
Mean (SD)	9.13 (0.73)	9.06 (0.71)	
**Midday sleep, n (%)**			
Yes	481 (81.8)	1079 (82.1)	
No	70 (11.9)	161 (12.3)	0.783
Missing	37 (6.29)	74 (5.63)	
Duration (hours), Mean (SD)	1.01 (0.52)	1.04 (0.54)	0.841
**Total daily sleep duration (hours)**			
Mean (SD)	10.1 (0.88)	10.1 (0.90)	0.470
**CSHQ Subscale score, mean (SD)**			
Bedtime resistance	16.1 (5.09)	15.4 (4.84)	0.005
Sleep onset delay	2.02 (1.02)	1.97 (0.99)	0.138
Sleep duration	2.18 (0.93)	2.11 (0.93)	0.223
Sleep anxiety	4.16 (2.40)	4.00 (2.24)	0.314
Night waking	4.19 (1.54)	4.25 (1.60)	0.199
Parasomnias	5.23 (1.90)	5.12 (1.80)	0.314
Sleep disordered breathing	1.51 (0.79)	1.51 (0.83)	0.318
Daytime sleepiness	9.32 (2.26)	9.29 (2.29)	0.949
Total score, mean (SD)	44.4 (9.93)	43.5 (9.55)	0.130
**Total time spent in near work (hours/week), mean (SD)**	22.1±10.1	22.6±11.3	0.115
**Total time outdoors (hours/week), mean (SD)**	14.0 (3.95)	14.7 (4.52)	0.001

* P value comparing the difference in sleep patterns and CSHQ scale scores between myopic and non-myopic children

**P-value based on categorical rather than continuous analysis

The score for "bedtime resistance" on the Children's Sleep Habits Questionnaire (CSHQ) was significantly higher for myopic (16.1 [5.09]) as compared to non-myopic (15.4 [4.84], P = 0.005) children, though scores for "Sleep onset delay," "Sleep duration," "Sleep anxiety," "Night waking," "Parasomnias," "Sleep-disordered breathing" and "Daytime sleepiness" did not differ between these groups (P > 0.10 for all). The mean total score for myopic children was did not differ significantly among children with myopia (44.4 [9.93]) compared to those without (43.5 [9.55], P = 0.130) (Table 2), as was the prevalence of an abnormal total CSHQ score (> = 41 points; myopic children 365/588 = 62.1%; non-myopic children 764/1314 = 58.1%, P = 0.107).

The average number of hours spent reading per week did not differ significantly between myopic (22.1 [10.1] hours/week) and non-myopic (22.6 [11.3] hours/week, P = 0.115) children, though time outdoors was significantly less for myopic (14.0 [3.95] hours/week) compared to non-myopic (14.7 [4.52] hours/week, P = 0.001) children. (Table 2)

In multi-level mixed effects logistic regression models of potential predictors of having myopia < = -0.5D in an eye, odds of myopia < = -0.50D increased with worse CSHQ score (Odds Ratio [OR] 1.01, 95% Confidence Interval [CI] [1.01, 1.02], P = 0.014) and more time spent in night-time sleep (OR 1.02, 95% CI [1.01, 1.04], P = 0.002), while male sex (OR 0.82, 95% CI [0.70, 0.95], P = 0.008) and time outdoors (OR = 0.97, 95% CI [0.95, 0.99], P = 0.011) were associated with less myopia. Age and near work were not significantly associated with myopia. (Table 3). In separate models (S1 Table, S2 Table, S3 Table), midday sleep time (P = 0.404) was not significantly associated with myopia, and total sleep duration (night-time + midday sleep) was not significantly associated (P = 0.199) when it was substituted for the night-time sleep variable.

**Table 3:** Logistic regression model of possible predictors of myopia < = -0.5D (Both eyes of all children are included, with the correlation between eyes adjusted for using multilevel logistic models.

	**Simple regression**		**Multiple regression** *	
	**Odds ratio (95% CI)**	**P**	**Odds ratio (95% CI)**	**P**
Age	0.79 (0.65, 0.97)	0.025	0.85 (0.68, 1.07)	0.175
Male Sex	0.82 (0.71, 0.93)	0.003	0.82 (0.70, 0.95)	0.008
Total CSHQ score	1.01 (1.00, 1.02)	0.038	1.01 (1.00, 1.02)	0.014
Night-time Sleep time (hours/week)	1.02 (1.00, 1.03)	0.021	1.02 (1.01, 1.04)	0.002
Total time spent in near work (hours/week)	0.99 (0.99, 1.00)	0.141	0.99 (0.99, 1.00)	0.062
Total time outdoors (hours/week)	0.97 (0.95, 0.99)	0.002	0.97 (0.95, 0.99)	0.011

*All potential predictors were included in the multiple regression model.

CHSQ = Children Sleep Habits Questionnaire

Models adjusting for group assignment in the trial gave essentially identical results to those described above. (S4 Table) When we used a cutoff of -1.0D in the definition of myopia, the results were similar (S5 Table): the association between total CSHQ score and myopia was still significant (P = 0.04), though the association between greater amounts of sleep and greater myopia risk was no longer significant (P = 0.08).

## Discussion

We found modest and somewhat contradictory evidence of an association between myopia and parentally-reported poor sleep in this population. Children with myopia had significantly higher bedtime sleep resistance sub-scores, and overall score on the CSHQ was significantly associated with myopia when adjusting for other predictors of refractive error. The magnitude of the difference remained modest, however, and the association was largely driven by the bedtime sleep resistance sub-score. Though our data do not allow us to address this hypothesis directly, it is possible that the association between bedtime resistance and myopia could be driven by greater academic pressure among myopic children. This would be consistent with reports of greater academic achievement among myopic children, [3] though subjective measures of perceived pressure and stress were not made in the current study.

Mean night-time sleep duration was actually longer among myopic children than non-myopic ones. It is possible that sleep duration was longer in myopic children as a marker of disordered sleep, as reflected in their somewhat higher CSHQ scores, but the modest observed difference (on the order of a few minutes per night) is unlikely to be consistent with an important etiologic role in the pathology of myopia. When total sleep duration (night-time + midday sleep) was considered, the association between sleep duration and myopia risk was no longer significant in univariate or multivariate analyses.

We are not aware of other published epidemiologic studies examining an association between myopia and poor sleep in children. A search of PubMed carried out in August 2013 for the words “myopia,” “refractive error,” “children” and “sleep” failed to identify any relevant documents.

As previously reported, [15] we found evidence that sleep disorders may be quite prevalent in Chinese children: the proportion of children with abnormal CSHQ scores (> = 41 points) in the current study was 1129/1902 (59.4%), and the mean score of 43.8 points was higher than previously recorded for similar-aged cohorts of American (38.7) and Chinese (42.1) children. [15] It has been suggested that the physiologic requirement for sleep among 9–10 year olds (the most common age range in this sample) is 10.0 hours/night, [28] and the average amount of sleep reported by similar-aged children in a recent study in the United States was 10 hours. [15] While nearly three-quarters of children (1344/1901 = 70.6%) in this cohort had an average parentally-reported night-time sleep duration of < = 9 hours, total sleep duration (including midday naps) was 10.1 hours. Evidence concerning the ability of midday sleep to compensate for inadequate night-time sleep as measured by work [29] and school-related [30] performance is contradictory, and there is little information on whether sleeping at midday affects circadian rhythms. Differences in sleep duration between Chinese and western children may in fact be less than previously reported [15] when accounting for midday sleep, though the biological and behavioral implications of the fact that some 10% of Chinese children's sleep occurs during the day rather than at night are not well understood. It may be that additional academic pressure and resulting anxiety in the major urban center of Guangzhou could explain the increased prevalence of sleep disorders in the current study compared to earlier Chinese reports, though our investigation was not designed to answer this question.

Normal circadian rhythms are described as being important to development of the human eye. [24–25] It seems plausible that disruption of these rhythms by disordered sleep might interfere with regulatory mechanisms controlling eye growth which underlie the emmetropization process, leading to refractive errors. Previous work has suggested the possibility that ambient lighting at night might be a risk factor for myopia, [31] though subsequent studies [32–4] failed to replicate the finding, and the observed association in the original study might have been confounded by parental factors. It is equally possible that modest associations between disordered sleep and myopia could be confounded by school schedule, as greater time in school appears to be a risk factor for poor sleep, [16] and may also be associated with myopia. [3] We sought to avoid such confounding by assessing time spent on schoolwork, and including it in our models, though we found no strong association between school-related near work and myopia in this cohort.

Our finding of more myopia among girls is consistent with previous studies among Chinese children. [33] Likewise, previous reports among children of Chinese [8–9, 11–12, 34] and other [10, 13–14] ethnicities have documented a protective effect of outdoor activity against myopia.

Strengths of the current study include the very high participation rate, use of a validated sleep questionnaire, [26–27] having collected separate data on both night-time and midday sleep duration, cycloplegic refraction of all children by an experienced optometrist, and ascertainment of other important potential determinants of refractive error such as near work and outdoor activity. Weaknesses include the fact that sleep data was available only by cross-sectional parental report (whereas architectural changes in the eye presumably occur over years), and that information on other potential determinants of myopia such as parental refraction were not available. Regarding the former, it is common in initial exploratory analyses to employ cross-sectional instruments requiring only inherently more-reliable short-term recall, under the assumption that recent behaviors are representative of longer-term trends. More time-consuming prospective techniques such as sleep diaries are available to pursue promising apparent relationships in future studies, though it is not clear from our initial results that such an investment is warranted in the case of sleep and myopia.

Despite these limitations, this paper is the first of which we are aware to examine a biologically and epidemiologically plausible association between sleep disturbances and myopia, both of which appear to be increasingly common among Chinese children. Though we found no evidence of a strong association between sleep and myopia, the high prevalence of disordered sleep warrants efforts to remediate the situation. Further research on any association between poor sleep and myopia is also recommended in different age groups and settings.

## Supporting Information

S1 Table Logistic regression model of possible predictors of myopia < = -0.5D (Both eyes of all children are included, with the correlation between eyes adjusted for using multilevel logistic models (added midday sleep time). (DOCX)

S2 Table Logistic regression model of possible predictors of myopia < = -0.5D (Both eyes of all children are included, with the correlation between eyes adjusted for using multilevel logistic models (With midday sleep time instead of night sleep time). (DOCX)

S3 Table Logistic regression model of possible predictors of myopia < = -0.5D (Both eyes of all children are included, with the correlation between eyes adjusted for using multilevel logistic models (With total sleep time instead of night sleep time). (DOCX)

S4 Table Logistic regression model of possible predictors of myopia < = -0.5D (Both eyes of all children are included, with the correlation between eyes adjusted for using multilevel logistic models, including adjustment for the intervention group). (DOCX)

S5 Table Logistic regression model of possible predictors of myopia < = -1.0D (Both eyes of all children are included, with the correlation between eyes adjusted for using multilevel logistic models. (DOCX)
