# Supplementary material for: Disordered Sleep and Myopia Risk among Chinese Children
Source: PLoS One. 2015 Mar 26;10(3):e0121796. doi: 10.1371/journal.pone.0121796 (PMC4374782; doi:10.1371/journal.pone.0121796)
Supplement: S1 Table — (DOCX) [file pone.0121796.s001.docx]

**Table S1 Logistic regression model of possible predictors of myopia <= -0.5D (Both eyes of all children are included, with the correlation between eyes adjusted for using multilevel logistic models (added midday sleep time)**

|  |  | **Simple regression** | |  | **Multiple regression*** | |
| --- | --- | --- | --- | --- | --- | --- |
|  |  | **Odds ratio (95% CI)** | **P** |  | **Odds ratio (95% CI)** | **P** |
| Age |  | 0.79 (0.65, 0.97) | 0.025 |  | 0.86 (0.68, 1.08) | 0.195 |
| Male Sex |  | 0.82 (0.71, 0.93) | 0.003 |  | 0.76 (0.65, 0.89) | <0.001 |
| Total CSHQ score |  | 1.01 (1.00, 1.02) | 0.038 |  | 1.01 (1.00, 1.02) | 0.042 |
| Night-time Sleep time (hours/week) |  | 1.02 (1.00, 1.03) | 0.021 |  | 1.02 (1.00, 1.03) | 0.029 |
| Midday Sleep time (hours/week) |  | 1.00 (0.98, 1.02) | 0.795 |  | 0.99 (0.97, 1.01) | 0.404 |
| Total time spent in near work (hours/week) |  | 0.99 (0.99, 1.00) | 0.141 |  | 0.99 (0.98, 1.00) | 0.068 |
| Total time outdoors (hours/week) |  | 0.97 (0.95, 0.99) | 0.002 |  | 0.98 (0.96, 1.00) | 0.063 |

*****All potential predictors were included in the multiple regression model.

CHSQ= Children Sleep Habits Questionnaire
