# Supplementary material for: Disordered Sleep and Myopia Risk among Chinese Children
Source: PLoS One. 2015 Mar 26;10(3):e0121796. doi: 10.1371/journal.pone.0121796 (PMC4374782; doi:10.1371/journal.pone.0121796)
Supplement: S5 Table — (DOCX) [file pone.0121796.s005.docx]

**Table S5 Logistic regression model of possible predictors of myopia <= -1.0D (Both eyes of all children are included, with the correlation between eyes adjusted for using multilevel logistic models**

|  |  | **Simple regression** | |  | **Multiple regression*** | |  |
| --- | --- | --- | --- | --- | --- | --- | --- |
|  |  | **Odds ratio (95% CI)** | **P** |  | **Odds ratio**  **(95% CI)** | **P** |  |
| Age |  | 0.98 (0.78, 1.21) | 0.772 |  | 1.01 (0.79, 1.30) | 0.936 |  |
| Male Sex |  | 0.92 (0.79, 1.07) | 0.276 |  | 0.89 (0.76, 1.05) | 0.178 |  |
| Total CSHQ score |  | 1.01 (1.00, 1.02) | 0.077 |  | 1.01 (1.00, 1.02) | 0.040 |  |
| Night-time Sleep time (hours/week) |  | 1.01 (0.99, 1.02) | 0.314 |  | 1.01 (1.00, 1.03) | 0.082 |  |
| Total time spent in near work (hours/week) |  | 1.00 (0.99, 1.00) | 0.288 |  | 1.00 (0.99, 1.00) | 0.285 |  |
| Total time outdoors (hours/week) |  | 0.96 (0.94, 0.98) | <0.001 |  | 0.97 (0.94, 0.99) | 0.003 |  |

*****All potential predictors were included in the multiple regression model.

CHSQ= Children Sleep Habits Questionnaire, higher score means more sleep disturbance.
